# ST9 MRSA strains carrying a variant of type IX SCC*mec* identified in the Thai community

**DOI:** 10.1186/1471-2334-13-214

**Published:** 2013-05-11

**Authors:** Aroonlug Lulitanond, Teruyo Ito, Shanshuang Li, Xiao Han, Xiao Xue Ma, Chulapan Engchanil, Aroonwadee Chanawong, Chotechana Wilailuckana, Netchanok Jiwakanon, Keiichi Hiramatsu

**Affiliations:** 1 Centre for Research and Development of Medical Diagnostic Laboratories, Faculty of Associated Medical Sciences, Khon Kaen University, Khon Kaen, Thailand; 2 Department of Infection Control Science, Graduate School of Medicine, Juntendo University, Tokyo, Japan; 3 Department of Bacteriology, Graduate School of Medicine, Juntendo University, 2-1-1 Hongo Bunkyo-ku, Tokyo 113-8421, Japan; 4 Research and Diagnostic Center of Emerging Infectious Diseases and Department of Microbiology, Faculty of Medicine, Khon Kaen University, Khon Kaen, Thailand; 5 Veterinary Research and Development (Upper Northeastern) Center, Khon Kaen, Thailand

**Keywords:** *S. aureus*, CA-MRSA, SCC*mec*, ST9, Livestock

## Abstract

**Background:**

Infections caused by methicillin-resistant *Staphylococcus aureus* (MRSA) in Thailand occur most frequently in healthcare facilities. However, reports of community-associated MRSA are limited.

**Methods:**

We characterized 14 MRSA isolates from outpatients (O-1 to O-14) by phenotypic and genotypic methods and compared them with 5 isolates from inpatients (I-1 to I-5). Thai MRSA isolates from a healthcare worker (N-1) and a pig (P-1) were also included as ST9 MRSA strains from other sources.

**Results:**

All MRSA isolates from the outpatients and inpatients were multidrug-resistant (resistant to ≥3 classes of antimicrobials). All of them except strains O-2 and I-3 carried type III SCC*mec* and belonged to *agr*I, coagulase IV, *spa* type t037 or t233, which related to ST239. The strain O-2 (JCSC6690) carried type IX SCC*mec* and belonged to *agr*II, coagulaseXIc, *spa* type t337 and ST9, whereas the strain I-3 carried a type III SCC*mec* and belonged to ST1429. Nucleotide sequence determination revealed that the type IX SCC*mec* element in strain O-2 was distinct from that in a Thai ST398 strain (JCSC6943) previously identified in 2011; nucleotide identities of *ccrA* and *ccrB* were 93 and 91%, respectively and several open reading frames (ORFs) at the joining regions were different. PCR experiments suggested that strain O-2 and N-1 carried similar SCC*mec* element, whereas that of strain P-1 was different, suggesting that distinct ST9-MRSA–IX clones might be spreading in this province.

**Conclusions:**

The SCC*mec*IX-ST9 MRSA clones of distinct SCC*mec* subtypes might have emerged in the Thai community and might also have disseminated into the hospital.

## Background

*Staphylococcus aureus* is an important human pathogen that causes a broad spectrum of infections, from mild to life-threatening. The organism becomes methicillin-resistant *Staphylococcus aureus* (MRSA) by acquiring the *mecA* gene. Expression of the *mecA* results in production of a special penicillin-binding protein, PBP2a, which has reduced affinity to β-lactam antibiotics [1] and confers resistance to all practically used β-lactam antimicrobials. The *mecA* gene is usually carried on a mobile genetic element called the staphylococcal cassette chromosome *mec* (SCC*mec*), which contains two main parts: the *ccr* gene complex (*ccr*) and the *mec* gene complex (*mec*). The *ccr* gene complex comprises *ccr* gene (s), *ccrA* and *ccrB,* or *ccrC,* which encode site-specific recombinases responsible for the mobility of SCC*mec,* and surrounding open reading frames (ORFs). The *mec* gene complex comprises the *mecA* gene, its regulatory genes, and insertion sequences upstream or downstream of the *mecA* gene [1]. SCC*mec* elements identified in *S. aureus* have been classified according to the combination of *ccr* allotypes with the *mec* gene complex: 11 types (I-XI) have been reported to date [2,3].

MRSA strains were regarded to be pathogens that cause hospital-acquired (HA) infections; however, MRSA infections in the community have been increasingly reported [4,5]. MRSA strains that colonized or caused infections in persons who meet the following criteria are considered to be CA-MRSA: healthy persons; person with no prior history of a healthcare-associated risk factors such as a recently (within the past year) hospitalized or had a medical procedure (such as dialysis, surgery, catheters) [5,6]. Recently, MRSA strains from livestock (e.g., pigs) or their products have emerged throughout Europe, America and Asia. Most of livestock-associated MRSA (LA-MRSA) strains belonged to clonal complex (CC) 398 as defined by multilocus sequence typing [7], although the other clones, e.g., CC5, CC9, CC30, and CC97, have been occurred [8].

Most of MRSA infection in Thailand was hospital-associated whereas CA-MRSA infection was very rare [9]. The hospital-associated MRSA strains usually carried types III or II SCC*mec* elements [10,11], while characteristics of CA-MRSA strains were not fully investigated. We aimed to characterize CA-MRSA strains disseminating in the Thai community compared with HA-MRSA strains from a teaching hospital in Thailand. Thai MRSA strains from other sources such as hospital staff, pig were also included and compared to a previously identified type IX SCC*mec* MRSA strain from a Thai veterinarian [12].

## Methods

### Bacterial strains

MRSA isolates (O-1 to O-14) from 14 outpatients at Srinagarind Hospital, Khon Kaen University, Thailand, were collected between September 2005 and March 2006: 11 isolates from skin or tissue infection samples and 3 from respiratory tracts. These strains met the category of CA-MRSA since they were isolated from samples collected from the outpatient, who did not admit to hospitals within a year. Five MRSA strains (I-1 to I-5) isolated from inpatients >72 hours after admission were also used as HA-MRSA control strains. In addition, 2 MRSA strains with type IX SCC*mec* (N-1 and P-1) were examined to investigate distribution of MRSA clone in the Thai community: the N-1 strain (isolated from a nasal swab of a nurse) was chosen from 19 *S. aureus* strains isolated during personnel surveillance in early 2006: the P-1 strain (from a pig with pneumonia) was chosen from 4 *S. aureus* strains collected at the Veterinary Research and Development Center of Upper Northeastern Thailand in 2006.

All isolates were identified by conventional coagulase and DNase tests. All of the MRSA strains from people were taken as part of standard care.

This study was conducted in accordance with the declaration of Helsinki and good clinical practice. It was approved by the Ethics Committee of Khon Kaen University (project number HE551393).

### Antimicrobial susceptibility test

The minimum inhibitory concentrations of 7 antimicrobials (vancomycin, cefazolin, oxacillin, cefoxitin, tetracycline, erythromycin and ofloxacin) (Sigma Chemical, St. Louis, USA) were determined using the agar dilution method described by the Clinical and Laboratory Standards Institute [13]. *S. aureus* ATCC29213 was used as a drug-susceptible standard strain.

### Multiplex PCR for SCC*mec* type

Chromosomal DNA was extracted using the DNeasy tissue Kit (Qiagen, Valencia, USA). Multiplex PCR for detection of the *mecA*, *ccr* gene complex type and *mec* gene complex class were performed using primer sets and conditions as per Kondo et al. [14]. MLEP (*mec* left extremity polymorphism) typing was performed to estimate the insertion of SCC*Hg* as per Ito et al. [15]. The *S. aureus* strains used as reference strains for SCC*mec* typing were: NCTC10442 (type I), N315 (type II), 85/2082 (type III), MR108 (type IV), JCSC3624 (type V), and HDE288 (type VI).

### PCR identification for heavy metal resistance genes and potential virulence genes

The *mer* operon and *copA* genes and genes encoding for virulent factors such as exfoliative toxin type a, b, d (*eta, etb, etd*), PVL (*lukS-PV, lukF-PV*) and TSST (*tst*) were detected by PCR methods [15-17].

### Molecular typing

Coagulase and *agr* typing was performed as per Sakai et al. [18] and Shopsin et al. [19], respectively. Typing of *spa* was performed as per Shopsin et al. [20]. The PCR products of the *spa* genes were then purified using High Pure PCR Product Purification kits (Roche Diagnostics, Indianapolis, USA) and used as DNA templates for sequencing by ABI PRISM 3100 (Applied Biosystems, Foster City, USA). The designation of the *spa* type was conducted using the RidomStaph Type program (http://www.ridom.de).

Pulsed-field gel electrophoresis (PFGE) was conducted as a method for genotyping. The MSSA strain NCTC8325 and MRSA strain N315 were used as reference strains for PFGE. The analysis of *Sma*I-digested chromosomal DNA was performed using a contour-clamped homogeneous electric field mapper system as per the manufacturer’s instructions (Bio-Rad, Hercules, USA), with a 48.5-kb ladder (Bio-Rad) as DNA-sized markers. The PFGE patterns for all of the isolates were analyzed using BioNumerics software (version 6.5; Applied Maths, Kortrijk, Belgium) and interpreted as per Tenover et al. [21].

Multilocus sequence typing (MLST) was conducted as per Enright et al. [22]. The alleles of the 7 loci were evaluated by comparing the sequences to those of the corresponding loci in the *S. aureus* MLST database (http://www.mlst.net). The sequence types (STs) were determined according to the combined patterns of the 7 alleles, while the clonal complexes were defined using the BURST program, based on related sequence types available on the MLST website.

### Determination of nucleotide sequences in and around the SCC*mec* element and *coa* gene

We determined the entire nucleotide sequences of type IX SCC*mec* carried by strain O-2 (JCSC6690) and nucleotide sequences of *coa* genes that could not be identified by PCRs using DNAs of three strains, O-2, N-1, and P-1. The DNA fragments of the SCC*mec* element from the strain O-2 and its genetics surrounding were amplified using long-range PCRs (list of primers sets in Additional file 1: Table S1). The region from Tn*916* to *copA* gene was amplified by using a constructed DNA template from the Fosmid Library Production Kit (Epicentre Biotechnologies, Madison, WI). The nucleotide sequences were determined by primer-walking. The sequences were analyzed for ORFs with the In Silico Molecular Cloning Program (IMC Load command) and compared with the sequence databases from the National Center for Biotechnology Information using the basic local alignment search tool, BLAST (National Library of Medicine, Bethesda, MD) for annotation and prediction of functions. The nucleotide sequences of the *coa* gene from strains O-2, N-1 and P-1 were determined as per Watanabe et al. [23].

### PCR identification of ORFs at J3 regions of type IX SCC*mec* elements

**Figure 1:**
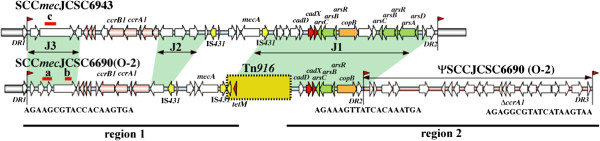
**Structural comparison of type IX SCC*mec *elements carried by strains O-2 and JCSC6943.** Structures of SCC*mec* are illustrated based on the nucleotide sequences deposited in databases DDBJ/EMBL/GenBank, under accession nos. AB505628 for JCSC6943, AB705452 (region 1, indicated by black bar) and AB705453 (region 2, indicated by black bar) for O-2. The red arrowhead indicates the location of ISS, and the nucleotide sequences are given below. ORFs are colored as follows: yellow, insertion sequences; red, *cad* operons; lawn green, genes for arsenical resistance operons; orange, *copB;* salmon, ORFs for *ccr* gene complexes. The three joining regions (J1, J2, and J3 regions) are indicated in green. The locations of DNA fragments amplified with three pairs of primers (a, b, and c) are indicated in red bars. Nucleotide sequences of primers used for amplification for three loci are listed in Additional file 1: Table S1.

The carriage of ORFs at the J3 region of SCC*mec*IX elements was examined using chromosomal DNAs of strains N-1 and P-1 with three sets of primers (sets a and b as used for the strain O-2 and set c as used for the strain JCSC6943) [12]. The locations and nucleotide sequences of the primers used are indicated in Figure 1 and Additional file 1: Table S1, respectively.

### Nucleotide sequence accession numbers

The respective nucleotide sequences of the two chromosomal regions containing the SCC*mec* element in strain O-2 were deposited in the DDBJ/EMBL/GenBank database under accession numbers AB705452 and AB705453. The nucleotide sequences of three *coa* genes were deposited in the DDBJ/EMBL/GenBank database under accession numbers AB742446 (strain O-2), AB742447 (strain N-1) and AB787218 (strain P-1).

## Results

### Characteristics of MRSA isolates

All outpatient and inpatient MRSA strains were multidrug-resistant with high MIC values to cefazolin, oxacillin, cefoxitin, tetracycline, erythromycin and ofloxacin (32 - >64 μg/ml). However, all of them were still susceptible to vancomycin.

None of the MRSA isolates carried the virulence-related genes tested*.* Thirteen out of 14 outpatient isolates and all 5 inpatient isolates carried a type 3 *ccr* gene and class A *mec* gene complex, although 12 out of 13 outpatient isolates and 4 out of 5 inpatient isolates also carried the *ccrC.* Further PCR experiments showed that these 16 strains contained mercury resistance gene (*mer*) and the extremity of SCC*Hg*, therefore, they were identified as type III SCC*mec* strains carrying SCC*Hg*[24]. In conclusion, 18 out of 19 strains carried type III SCC*mec*. The remaining strain, O-2, carried *ccrA1B1* genes and class C2 *mec* gene complex, which was categorized to type IX SCC*mec*.

All isolates from both sources were of the *agr* group I and coagulase type IV except for strain O-2 that belonged to *agr* group II. Since the coagulase type of strain O-2 could not be determined neither by a genetic method using multiplex PCRs nor by serological typing based on a coagulation inhibition, its coagulase nucleotide sequences were determined, and classified into type XIc, a novel coagulase subtype. The majority of type III SCC*mec* strains belonged to *spa* type t037 except for strains O-3 and I-3, which belonged to *spa* type t233 and nontypeable, respectively. No amplification product of *spa*-typing was seen when used chromosomal DNA of strain I-3 as DNA templates. MLST genotypes of 5 *spa* t037 isolates, which were chosen based on their unique PFGE banding patterns, were identified as the ST239, whereas those of strain O-3 and I-3 were ST239 and ST1429 respectively. The strain O-2 belonged to *spa* type t337 and ST9. The characteristics of strain O-2 were distinct from other 18 strains.

**Figure 2:**
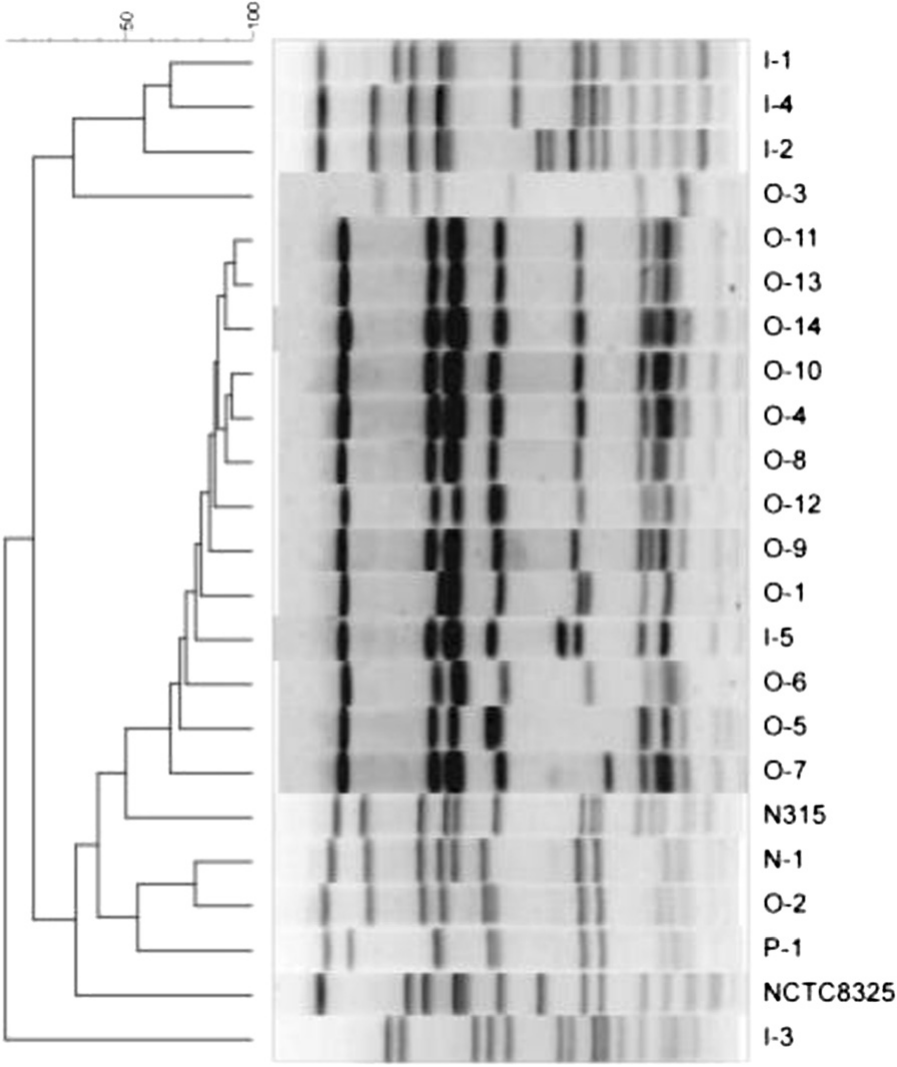
**Dendrographic analysis of Pulsed-field Gel Electrophoresis banding patterns of *Sma***I***-*digested chromosomal DNAs of MRSA strains from human and a pig.** PFGE patterns of 14 MRSA strains from outpatients (O-1 to O-14), 5 MRSA strains from inpatients (I-1 to I-5), an MRSA strain from a healthcare worker (N-1) and an MRSA strain from a pig (P-1) were compared. Curve-based phylogenetic tree using Ranked Pearson Correlation was generated using BioNewmerics.

The PFGE profiles revealed that 12 out of 14 outpatient isolates showed similar banding patterns with 1–2 different bands, while strains O-2 and O-3 had a distinct DNA profile (Figure 2). Three of the five inpatient isolates showed closely related PFGE patterns and strain I-3 belonging to ST1429 presented a distinct pattern, whereas the band pattern of strain I-5 was related to the outpatient strains (Figure 2).

### Structure of type IX SCC*mec* in strain O-2 (SCC*mec*O-2)

The nucleotide sequences of the two long regions containing SCC*mec* element in the strain O-2 were determined. The first 25 kb region contained a class C2 *mec* gene complex, a type 1 *ccr* gene complex and the downstream end of a conjugative transposon, Tn*916*. The second 32 kb region started from the upstream region of Tn*916* and ended at the extremity of an SCC. The overall structure of the approximately 70-kb region from the strain O-2 containing the SCC*mec* element and an SCC without *ccr* gene (ΨSCC) is illustrated in Figure 1 and the ORFs locating in these regions are listed in Table 1.

**Table 1:** ORFs of SCC***mec*** in the ***S. aureus ***strain O-2 compared to ***S. aureus ***JCSC6943 and ***S. haemolyticus ***JCSC1435

**ORFs**	**Genes**	**Description of products**	**% identity to type IX SCC *mec * in *S. aureus *JCSC6943**^**a**^	**% identity to *S. haemolyticus *JCSC1435**
KK01	*orfx*	hypothetical protein	97	80
KK02		hypothetical protein, similar to modification methylase LlaFI	<50	51.8
KK03		hypothetical protein, similar to[truncated]modification methylase LlaFI	<50	50.3
KK04		hypothetical protein, similar to restriction enzyme LlaFI	<50	<50
KK05		hypothetical protein	52	83
KK06		hypothetical protein	65	86.3
KK07		hypothetical protein	78	61.9
KK08		hypothetical protein	85	83
KK09		hypothetical protein	<50	51.6
KK10	*ccrB1*	cassette chromosome recombinase B1	91	50
KK11	*ccrA1*	cassette chromosome recombinase A1	93	<50
KK12		hypothetical protein	81	51.1
KK13		hypothetical protein	61	<50
KK14		hypothetical protein	95	<50
KK15	*tnp*	IS*431* transposase	97.5	99.1
KK16		[truncated]putative HMG-CoAsynthase	100	83
KK17		Glycerophosphoryl diester phosphodiesterase	100	100
KK18		hypothetical protein	100	100
KK19	*mecA*	penicillin-binding protein 2'	100	100
KK20	*tnp*	transposase for IS*431*	100	98
KK21	*tnp*	Tn*916*, transposase	<50	<50
KK22		Tn*916*, hypothetical protein	<50	<50
KK23		Tn*916*, transcriptional regulator	<50	50
KK24	*tetM*	tetracycline resistance protein TetM	<50	<50
KK25		hypothetical protein	<50	52.5
KK26	*cadD*	cadmium binding protein	96	94
KK27	*cadX*	cadmium resistant accessory protein	96	77
KK28	*arsC*	arsenate reductase	93	82
KK29	*arsB*	arsenic efflux pump protein	88	73
KK30	*arsR*	arsenical resistance operon repressor	100	97
KK31	*copA*	copper-exporting ATPase	98	67
KK32		putative lipoprotein	92	98

ORFs, KK1-KK24 and KK25-32 are from nucleotide sequences deposited at the DDBJ/EMBL/ GenBank databases under accession nos. AB705452 and AB705453, respectively.

^a^ % identities of less than 50% were expressed as <50%.

Three integration site sequences (ISS) comprising directly repeated (DR) sequences were identified: the first ISS, at the 3′ end of *orfX*; the second ISS, at the downstream of the *copB* gene on the 32-kb fragment; and the third ISS, at the end of the 32 kb fragment (Figure 1). The SCC*mec*O-2 was estimated to be 43 kb in size, in which the Tn*916* was integrated. At the downstream of the SCC*mec*, an SCC encoding truncated *ccrA1* had 83% nucleotide identity with that of type I SCC*mec* in strain NCTC10442.

We then compared the structure of SCC*mec*O-2 to a previously reported type IX SCC*mec* of strain JCSC6943 [12]. The class C2 *mec* gene complexes of both strains were highly identical, four ORFs around the *mecA* gene of the strain O-2 had 100% identity with SCC*mec*JCSC6943*.* The *ccr* gene complexes of both strains were located upstream to the *mec* gene complex. The nucleotide sequences of the *ccrA1B1* in SCC*mec*O-2 showed 91–93% identities with those of SCC*mec*JCSC6943 and 91–92% identities with those of the type I SCC*mec* in strain NCTC10442.

The ORFs at J3 region of SCC*mec*O-2 which encoded for hypothetical proteins related to modification methylase and restriction enzymes were less similar to those of the JCSC6943 (Table 1). At the J1 region of both SCC*mec* elements, the ORFs encoding resistance genes for heavy metals (*e.g.,* cadmium and copper) were present. But a truncated copy of Tn*916* encoding tetracycline resistance gene (*tetM*) was identified only in the SCC*mec*O-2 (Table 1).

### Comparisons of type IX SCC*mec* MRSA isolates

The characteristic of type IX SCC*mec* MRSA strains, N-1 and P-1, were compared with strain O-2. All 3 strains belonged to the same *agr* type and ST type (Table 2). Nucleotide sequences of *coa* genes of these three strains were exactly identical, which were classified into type XIc. However, their *spa* types were not totally identical. Strain P-1 belonged to *spa* type t044, whereas strains N-1 and O-2 belonged to the same *spa* type t337. These three isolates showed closely related PFGE patterns with 2–4 different bands (Figure 2).

**Table 2:** Characteristics of MRSA strains tested in this study

**Isolate no.**^**a**^	**Origin of specimen**	**Disease**	**Minimum inhibitory concentration**^**b**^**(μg/mL)**							***ccr* gene type**	***mec *gene complex class**	***mer *operon**	***copA***	***agr *type**	**Coagulase type**	***spa *type**	**MLST (ST)**
			**Van**	**Cefa**	**Oxa**	**Cefox**	**Tetra**	**Erythro**	**Ofx**								
Isolates from outpatients (n = 14)																	
O-1	Pus Left thigh (Human)	Accident, infected wound	2	> 64	> 64	> 64	32	> 64	> 64	*ccrA3B3*	A	-	-	I	IV	t037	ST239
O-2	Pus Leftfoot (Human)	Atopic dermatitis	1	32	32	32	> 64	> 64	> 64	*ccrA1B1*	C2	-	+	II	XIc	t337	ST9
O-3	Wound (Human)	Electrical burn	2	> 64	> 64	> 64	64	> 64	> 64	*ccrA3B3ccrC*	A	+	-	I	IV	t233	ST239
O-4	Pus (Human)	NA	2	> 64	> 64	> 64	> 64	> 64	> 64	*ccrA3B3ccrC*	A	+	-	I	IV	t037	NT
O-5	Pus (Human)	Infected wound	1	> 64	> 64	> 64	> 64	> 64	> 64	*ccrA3B3ccrC*	A	+	+	I	IV	t037	NT
O-6	Tracheal suction (Human)	NA	2	> 64	> 64	> 64	> 64	> 64	> 64	*ccrA3B3ccrC*	A	+	-	I	IV	t037	ST239
O-7	Sputum (Human)	Pulmonary TB	2	> 64	> 64	> 64	> 64	> 64	> 64	*ccrA3B3ccrC*	A	+	-	I	IV	t037	NT
O-8	Wound (Human)	Accident, head injury	2	> 64	> 64	> 64	64	> 64	> 64	*ccrA3B3ccrC*	A	+	-	I	IV	t037	NT
O-9	Wound (Human)	Accident, infected wound	2	> 64	> 64	> 64	> 64	> 64	> 64	*ccrA3B3ccrC*	A	+	-	I	IV	t037	NT
O-10	Pus Left cheek (Human)	NA	1	> 64	> 64	> 64	64	> 64	> 64	*ccrA3B3ccrC*	A	+	-	I	IV	t037	NT
O-11	Pus scalp (Human)	Chronic scalp impetigo	2	> 64	> 64	> 64	64	> 64	> 64	*ccrA3B3ccrC*	A	+	-	I	IV	t037	NT
O-12	Tissue (Human)	Gas gangrene	2	> 64	> 64	> 64	32	> 64	> 64	*ccrA3B3ccrC*	A	+	-	I	IV	t037	NT
O-13	Sputum (Human)	NA	2	> 64	> 64	> 64	64	> 64	> 64	*ccrA3B3ccrC*	A	+	-	I	IV	t037	NT
O-14	Pus Lt thigh (Human)	Accident, infected wound	2	> 64	> 64	> 64	> 64	> 64	> 64	*ccrA3B3ccrC*	A	+	-	I	IV	t037	NT
Isolates from in patients (n = 5)																	
I-1	Blood (Human)	NA	2	> 64	> 64	> 64	> 64	> 64	> 64	*ccrA3B3*	A	+	-	I	IV	t037	NT
I-2	Blood (Human)	NA	1	> 64	> 64	> 64	> 64	16	> 64	*ccrA3B3ccrC*	A	+	-	I	IV	t037	NT
I-3	Blood (Human)	NA	1	> 64	> 64	> 64	8	> 64	> 64	*ccrA3B3ccrC*	A	+	+	I	IV	UD^c^	ST1429
I-4	Sputum (Human)	NA	2	> 64	> 64	> 64	> 64	> 64	> 64	*ccrA3B3ccrC*	A	+	-	I	IV	t037	ST239
I-5	Sputum (Human)	NA	2	> 64	> 64	> 64	32	> 64	> 64	*ccrA3B3ccrC*	A	-	-	I	IV	t037	ST239
An isolate from healthy worker (n = 1)																	
N-1	Nasal swab (Human)	colonize	1	> 64	64	32	64	1	> 64	*ccrA1B1*	C2	-	+	II	XIc	t337	ST9
An isolate from pig (n = 1)																	
P-1	Lung (pig)	pneumonia	1	> 64	> 64	64	> 64	64	> 64	*ccrA1B1*	C2	-	+	II	XIc	t044	ST9

^a^ O, outpatient; I, inpatient; N, nurse; P, pig; NA, information was not available.

^b^ Van, vancomycin; Cefa, cefazolin; Oxa, oxacillin; Cefox, cefoxitin; Tetra, tetracycline; Erythro, erythromycin; Ofx, ofloxacin.

^C^ ND,Undetermined; because no PCR product was amplified.

The carriage of genes in strains N-1 and P-1 examined by using primer’s pairs (a, b and c) showed that strains O-2 and N-1 were positive in PCRs using primer pairs a and b, while strain P-1 was negative with all three primer’s pairs. Although strains N-1 and P-1 were positive in PCRs identifying *tetM* and *copB* genes, the locations of these genes were not examined yet.

## Discussion

### Characteristics of type IX SCC*mec* in strain O-2

The SCC*mec*O-2, which was located on the downstream region of the *orfX,* was classified into type IX. It had type I *ccr* gene complex carrying *ccrA* and *ccrB* genes that were classified into *ccrA1* and *ccrB1* based on the criteria suggested by IWG-SCC [2]. The class C2 *mec* gene complex of SCC*mec*O-2 was very similar to that of SCC*mec*JCSC6943 [12], the *ccr* gene complex was located between *orfX* and *mec* gene complex. Furthermore, a number of ORFs encoding genes associated with heavy metal resistance (i.e., *cadD, cadX, arsC, arsB, arsR* and *copB*) were also found and showed 92-100% nucleotide identities to those of the SCC*mec*JCSC6943. However, a number of ORFs were absent in the SCC*mec*JCSC6943, e.g., hypothetical proteins related to restriction-modification system at J3 region and a conjugative transposon Tn*916* encoding *tetM* at J1 region. The transposon Tn*916* was located just downstream of the class C2 *mec* gene complex and the extremity of Tn*916* including *int* gene was truncated. The transposition of Tn*916* was not site-specific. We presume that Tn*916* transposed firstly into the SCC element, then the class C2 *mec* gene complex was integrated to the SCC harboring Tn*916* by disrupting its extremity.

The class C2 *mec* gene complex was formerly reported predominantly in *S. haemolyticus*[1]*.* Comparison of the ORFs of SCC*mec*O-2 and those of *S. haemolyticus* strain JCSC1435 revealed that their heavy metal-resistant genes (e.g., *cadD, cadX, arsC, arsB, arsR, copB*) had 67-97% identities. Three ORFs at the J3 region had more than 80% identities with those of the *S. haemolyticus* strain JCSC1435 [25]. These data suggested that although the SCC*mec*O-2 and SCC*mec*JCSC6943 were classified into type IX, they might have evolved independently. Further study will clarify whether any staphylococcal strains such as the *S. haemolyticus,* might serve as reservoirs for SCC or SCC*mec* elements (Table 1).

### MRSA clones in Thailand

MRSA infections in the hospitals have been a big concern in Thailand. So far the HA-MRSA strains have been analyzed, ST239-SCC*mec*III clone was the major, although ST5-SCC*mec*II clone was identified as the minor one [10,11]. On the other hand, CA-MRSA in Thailand was reported to be very rare [9]. The incidence of CA-MRSA was undetermined because most outpatients with non severe infections were treated empirically without collecting samples for culture [26]. Most of the MRSA isolates from outpatients in the present study were ST239-SCC*mec*III-*spa* type t037, which was the dominant clone reported in Asian countries except for Korea and Japan [10]. CA-MRSA has been reported to be susceptible to a wide range of non-β-lactam antibiotics, low oxacillin MIC, but our tested strains were multidrug-resistant to non-β-lactam antimicrobials similar to HA-MRSA strains [27].

Our results were consistent with the report from ANSORP that ST239-SCC*mec*III and ST5-SCC*mec*II MRSA clones were found among CA-MRSA isolates from patients in Asian countries (*e.g.*, Vietnam, Thailand, Sri Lanka, Taiwan, and Hong Kong), without any risk factors for HA-MRSA infection [5]. The results of our PFGE experiment suggested that ST239*–*SCC*mec*III strains disseminated in the community were rather clonal and distinct from those in the hospital. Further detailed study will be needed to clarify this hypothesis.

### A novel MRSA clone in the Thai community

It has been reported that CA-MRSA strains tend to carry types IV or V SCC*mec* element and belonged to particular genotypes. The distribution of CA-MRSA clones varied by geographical area: the ST1-SCC*mec*IV and ST8-SCC*mec*IV strains (called as USA400 and USA300 based on PFGE patterns) in the USA; the CC80-SCC*mec*IV strains in Europe and Africa; ST22-SCC*mec*IV and SCC*mec*V- ST772 strains in India; ST30-SCC*mec*IV and ST93-SCC*mec*IV strains in Australia; and CC59-SCC*mec*V strains in Asia [28,29]. Interestingly, most of these CA-MRSA strains carried the Panton Valentine Leukocidin (PVL) gene that encodes a necrotizing cytotoxin, which may be responsible for the invasiveness and virulence of the organism [30]. Recently livestock associated MRSA (LA-MRSA) have emerged in the community and spread into hospitals [31-34]. The majority of LA-MRSA strains belonged to ST398, while strains belonged to other STs, e.g. ST9, has also been reported.

In this study, we analyzed three ST9 MRSA strains carrying SCC*mec*IX, the same SCC*mec* type identified in an ST398-MRSA isolated from a Thai veterinarian (strain JCSC6943) [12].

The strain O-2 was isolated in March 2006, from a young boy with underlying atopic dermatitis and suffering from chronic impetigo of the left foot. He had not been admitted to hospital in the prior year. Therefore, we regarded the strains as CA-MRSA and as a novel MRSA clone emerged in the Thai community. We explored the clone and identified 2 strains, one (strain P-1) from a diseased pig with pneumonia in the same province and the other (strain N-1) from a nasal swab of a nurse isolated during hospital-personnel surveillance in the same year as the first case. The ST9-MSSA has occasionally been isolated from healthy human carriers [35] and has been able to colonize and be transmitted between humans and pigs as it has also been isolated from pigs and workers on pig farms [36,37]. Recently ST9-SCC*mec*IX strains have been isolated from swine in Thailand [38,39]. In the present study, 2 isolates of ST9-SCC*mec*IX were from humans. To our knowledge, this is the first report of ST9-SCC*mec*IX strain from humans (a patient and a healthy healthcare worker) and a diseased pig in Thailand. Unfortunately, information regarding any animal contact by these persons or their families was not available. The ST9-SCC*mec*IX strain would belong to a newly identified CA-MRSA clone disseminating in the Thai community, although no isolate harbored any potential virulence genes including the *pvl* gene, which is usually found in CA-MRSA strains.

The detailed comparison of 3 ST9-SCC*mec*IX strains: O-2, N-1, and P-1, suggested that strains isolated from human and pig were not exactly identical. Their PFGE patterns were not totally identical but they were closely related, in contrast, that of ST398 strain JCSC6943 showed a non-typeable pattern (absence of DNA fragment). The *spa* type of strains O-2 and N-1 belonged to t337, whereas strain P-1 belonged to t044. Recently, MRSA isolates from pigs and pork in Thailand carrying the SCC*mec*IX-*spa* type t337 have been reported [38,39]. The ST9-*spa* type t899 MRSA and MSSA were recently isolated from dust samples taken from Chinese pig farms [40]. Most of these isolates were resistant to various classes of antimicrobials such as ß-lactams, macrolides and tetracycline, which is consistent with the strains O-2, N-1 and P-1. However, those strains belonged to the SCC*mec* type III.

PCR identification of J regions in strains O-2 and N-1 suggested that they carried the same subtype of type IX SCC*mec*, which was distinct from that of strain P-1 and JCSC 6943. On the other hand, strain P-1 was assumed to carry a novel subtype of type IX SCC*mec*, since it was distinct from that of strains O-2 and JCSC6943. We presume that livestock, e.g., pigs, might be the reservoir of ST9-*S. aureus* in Asian countries. The ST9-*S. aureus* strain might become an MRSA by acquiring SCC*mec* elements from different sources, since ST9-SCC*mec*III strains were identified in China [40]. Identification of several subtypes of type IX SCC*mec* elements suggested that such elements might come from elsewhere and integrated into ST9 strains. In this study, it could not be concluded that SCC*mec*IX strains from human were derived from pig, since strain P-1 was not identical to strains O-2 and N-1. However, SCC*mec*IX strains with the same *spa* type (t337) have been identified from pigs (38, 39). Further study of these isolates will clarify whether ST9 MRSA strains isolated from human might be derived from livestock and disseminated in the Thai community.

## Conclusions

ST9-SCCmecIX MRSA strains from humans (a patient and a healthy healthcare worker) and a diseased pig in Thailand were reported as a newly identified CA-MRSA clone disseminating in the Thai community.

## Competing interests

All authors declare that they have no competing interests.

## Authors’ contributions

AL participated in study design, data collection and interpretation, drafted and revised the manuscript. TI contributed to study design and revised the manuscript. SSL, HX and MXX completed all data analyses. CE, AC, CW, and NJ participated in data collection and analyses. KH contributed to study design and revised the manuscript. All authors read and approved the final manuscript.

## Pre-publication history

The pre-publication history for this paper can be accessed here:

http://www.biomedcentral.com/1471-2334/13/214/prepub

## Supplementary Material

Additional file 1: Table S1 Sequences of oligonucleotide primers for long range PCR. Click here for file
